# Vegetation Analysis and Environmental Relationships of Qatar’s Depression Habitat

**DOI:** 10.3390/plants14121807

**Published:** 2025-06-12

**Authors:** Ahmed Elgharib, María del Mar Trigo, Mohamed M. Moursy, Alaaeldin Soultan

**Affiliations:** 1 Department of Botany and Plant Physiology, University of Malaga, Campus de Teatinos s/n, 29071 Malaga, Spain; aerox@uma.es; 2 Botany and Microbiology Department, Faculty of Science Al-Azhar University, Cairo 11884, Egypt; mmmoursy@azhar.edu.eg; 3 Swiss Ornithological Institute, 6204 Sempach, Switzerland

**Keywords:** depression, floristic diversity, canonical correspondence analysis, TWINSPAN, soil–vegetation relationships, phytogeographical analysis

## Abstract

Despite Qatar’s depressions being ecologically significant for biodiversity in arid desert regions, they remain poorly studied. This study aimed at assessing the floristic diversity of Qatar’s depression habitat and examining the key environmental drivers shaping vegetation patterns. We applied multivariate analyses, including Canonical Correspondence Analysis (CCA) and Two-Way Indicator Species Analysis (TWINSPAN), to understand the environmental factors that shape vegetation communities and classify the depression sites. A total of 139 plant species from 35 families were recorded from 26 depression sites across Qatar. Both therophytes and chamaephytes were the dominant life forms. Biregional chorotypes were the most prevalent among phytogeographical groups. CCA indicated that grazing pressure, latitude, nitrogen concentration, clay content, and soil pH were among the variables that influenced the vegetation patterns of depressions, while longitude and soil carbon content showed marginal significance in explaining the observed floristic variation. TWINSPAN classified the sites into four distinct clusters, each associated with specific indicator species and habitat conditions. Northern depressions supported higher species richness compared to central and southern depressions, which are dominated by sandy soils and experience intensive grazing patterns that reduce the floristic diversity and limited regeneration of key shrubs such as *Vachellia tortilis* (Forssk.) Galasso & Banfi. This study helps fill a critical knowledge gap about Qatar’s depression habitat, enhancing efforts to conserve these vulnerable ecosystems, identify ecological threats, and better understand patterns of species distribution across arid landscapes.

## 1. Introduction

Qatar’s biodiversity is shaped by extreme climatic conditions, including high temperatures, limited and erratic rainfall, and nutrient-poor soils [1]. Despite the predominant harsh environmental conditions, depression, locally known as “Rawdat”, harbours a wide array of native plant species [2]. The depressions are characterised by their ability to retain moisture and accumulate organic matter, which results in creating microhabitats that support a variety of perennial and annual plant species [3]. The unique combination of soil properties, hydrological processes, and climate variability makes depressions critical for the survival of vegetation in Qatar’s desert ecosystem and essential for local biodiversity [2,4].

Soil plays a significant role in shaping the floristic diversity of depression habitat [2,5,6]. For instance, depressions in the northern part of the country are characterised by clay-rich soils, which support the survival of tree species such as *Ziziphus nummularia* (Burm.f.) Wight & Arn. and *Vachellia tortilis*, while sandy depressions (mostly in the southern part of the country) are dominated by drought-resistant shrub species such as *Panicum turgidum* Forssk. and *Cyperus conglomeratus* Rottb. [2,7]. Additionally, seasonal rainfall plays a vital role in shaping these ecosystems by promoting ephemeral plant growth and sustaining perennial species [2,3]. After rainfall events, depressions can temporarily flood, holding moisture long after the surrounding desert has dried out [2,8]. This allows depressions to harbour a wide range of vegetation types, including trees, shrubs, grasses, and herbs that provide potential habitat for wildlife and contribute to ecosystem functioning [1,2,3,9].

Despite their high ecological importance, depression habitat faces multiple anthropogenic threats that might deteriorate the sustainability of such habitats [10]. For instance, overgrazing by livestock can deplete vegetation cover, disrupt seed banks, compact the soil, and reduce the depression’s ability to retain water [6,11]. Off-road driving is another threat that destroys vegetation directly and exacerbates soil erosion [12]. Mining activities and military operations further degrade depression habitat by altering soil profiles and hydrology [13]. Climate change intensifies these impacts by reducing rainfall frequency, increasing temperatures, and degrading soil properties [14]. Further, introduced plant species also pose a significant threat by outcompeting native flora and altering ecological processes [15].

Yet, compared to the neighbouring countries such as Saudi Arabia [16,17,18] and Kuwait [19], depression habitat remains poorly studied in Qatar, where there is limited information on its floristic diversity, species composition, and the factors shaping vegetation patterns. Over the last decades, limited work has been conducted to assess the floristic diversity of the depression habitat of Qatar [1,2,3,4,20]. This knowledge gap hinders effective conservation efforts for this key habitat. To address this gap, this study aims to quantify the floristic diversity of depression habitat across Qatar and assess the environmental drivers, particularly soil variables, influencing the floristic diversity. To this end, we conducted an intensive field survey to sample the depression habitat across the country. We applied advanced multivariate techniques, including Canonical Correspondence Analysis (CCA) and Two-Way Indicator Species Analysis (TWINSPAN), to study the relationships between vegetation communities and environmental variables and to classify the depression sites based on their similarity in species composition. The findings of this study will offer valuable insights into the dynamics of arid ecosystems, enhancing our understanding of vegetation composition, prevailing threats, and soil types associated with depression habitat.

## 2. Results

### 2.1. Floristic Composition

**Figure 1:**
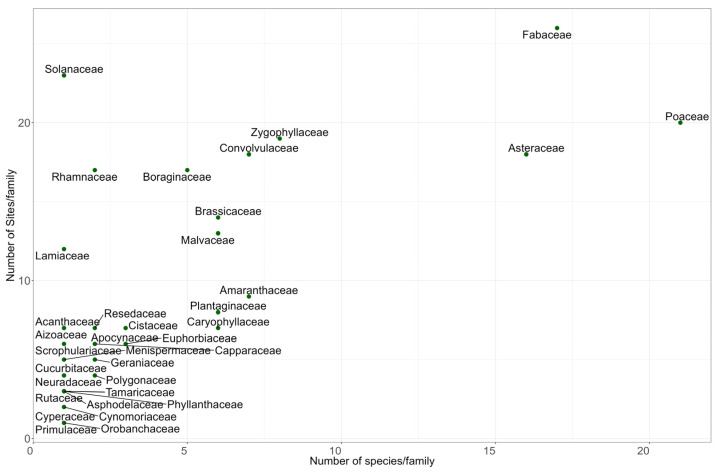
Plant species families and species richness across the study area.

A total of 139 species belonging to 35 families were recorded from 26 sites during the field survey. Of them, 81 were perennials and 58 were annuals. The most prevalent families are Poaceae, with 21 species, followed by Fabaceae and Asteraceae, with 17 and 16 species, respectively (Figure 1 and Table S1), while the remaining 32 families are poorly represented, the majority having single species. In the species-rich families such as Poaceae and Fabaceae, the northern sites are characterised by high richness compared to the central and southern sites (Figure 1 and Table S1).

### 2.2. Life Forms in Qatar’s Depression Habitat

**Figure 2:**
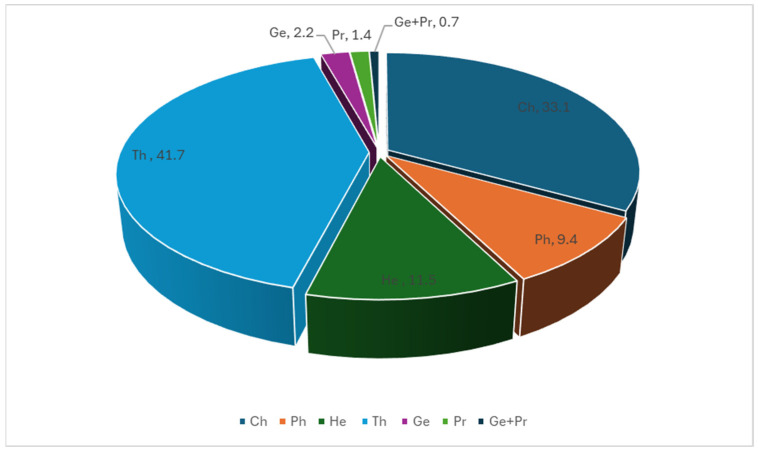
Life form spectrum of recorded species in Qatar’s depression habitat. The numbers correspond to the percentage of the life forms. Life form using classification as follows: Ch = chamaephytes; Ge = geophytes; He = hemicryptophytes; Pr = parasites; Ph = phanerophytes; Th = therophytes.

Our analysis showed that the therophytes (Th) were the most dominant life form, with 58 species (ca. 42%), followed by chamaephytes (Ch), comprising 46 species (33%), whereas geophytes (Ge) and parasites (Pr) were the least frequent life forms, represented by only 3 (ca. 2%) and 2 species (ca. 1.4%), respectively (Figure 2 and Table S1).

The regional distribution of life forms shows notable variations across the study area. Therophytes are most abundant in the northern region (41 species) compared to 22 in the south and 16 in the centre. Chamaephytes exhibit a higher frequency in both northern and southern regions (34 species each) compared to the central region (27 species).

### 2.3. Phytogeographical Affinities

The chorological analysis categorised the 139 recorded plant species into three phytogeographical groups: monoregional, biregional, and pluriregional (Table 1 and Table S1). Biregional chorotypes were the most represented, comprising 71 species (47.3% of the total flora), in which the Irano-Turanian/Saharo-Arabian (27 species) was predominant, followed by Mediterranean/Saharo-Arabian (15 species) and Sudanian/Saharo-Arabian (9 species). Monoregional chorotypes were the second most represented group, consisting of 41 species (27.4%), in which the Saharo-Arabian region was predominant, representing 26 species (17.3%), followed by the Sudanian region with 6 species, while pluriregional chorotype was the least represented, consisting of 27 species (18.0%) with different combinations such as Irano-Turanian/Mediterranean/Saharo-Arabian, as well as less commonly represented distribution types such as Pantropical (5 species), Cosmopolitan (4 species), and Palaeotropical (1 species).

Our results show a regional variation in the floristic composition of the depressions, where the northern part is dominated by the Saharo-Arabian chorotypes with significant representation from the Irano-Turanian/Saharo-Arabian and Mediterranean/Saharo-Arabian groups. The southern part exhibited a similar pattern with slightly fewer Saharo-Arabian and biregional representatives, whereas, the central part displayed lower overall diversity but retained a balanced representation of key chorotypes.

### 2.4. Vegetation Analysis

**Figure 3:**
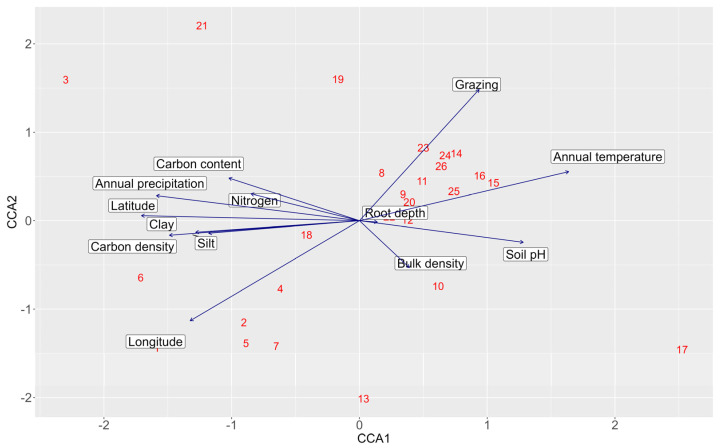
The bi-plot of CCA with environmental variables. The numbers refer to the depression site, and the arrows show the direction and the magnitude of the corresponding environmental variables.

The Canonical Correspondence Analysis (CCA) was conducted to examine how environmental, climatic, and spatial variables influence species composition across the study area. The CCA model explained a total inertia of 6.238, with ca. 58% of the variance constrained by the included environmental variables. The first two canonical axes (CCA1 and CCA2) explained ca. 15.5% and ca. 14.5% of the constrained variance, respectively, cumulatively accounting for ca. 30% of the total constrained variation. CCA1 represented a major spatial and edaphic gradient, driven by strong negative loadings for latitude, clay, and carbon density (−0.84, −0.63, and −0.73, respectively), and positive loadings for soil pH (+0.63) and annual mean temperature (+0.80). CCA2 captured variation primarily associated with grazing pressure (+0.73) and longitude (−0.56) (Figure 3). Permutation tests (999 permutations) revealed that several variables significantly explained variation in species composition. Grazing pressure (F = 2.01, *p* = 0.001), latitude (F = 1.56, *p* = 0.007), nitrogen (F = 1.58, *p* = 0.006), clay content (F = 1.37, *p* = 0.045), and soil pH (F = 1.38, *p* = 0.038) were statistically significant, while longitude (*p* = 0.055) and carbon content (*p* = 0.096) were marginally significant in explaining vegetation patterns.

### 2.5. Alpha Diversity

**Figure 4:**
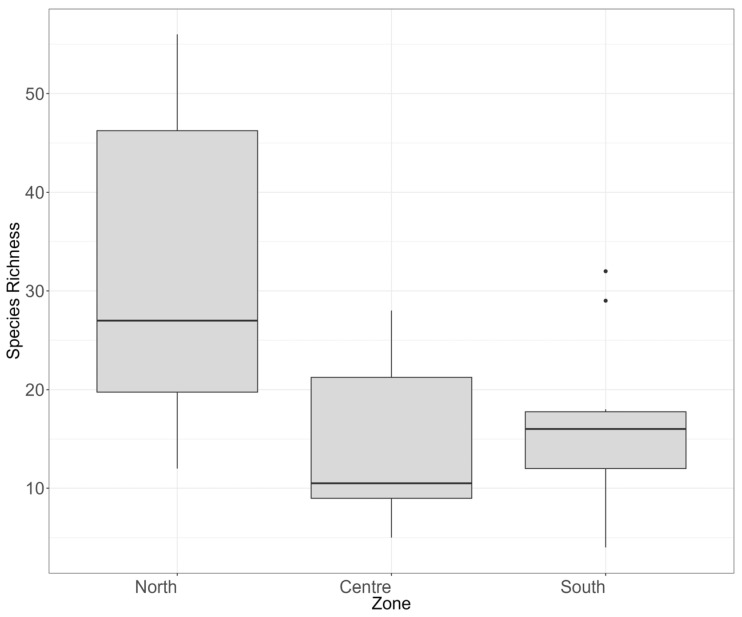
Species richness across the zones (north, south, and centre) of Qatar’s depression habitat. The horizontal line indicates the median, the whiskers extend to the minimum and maximum non-outlier values, and the dots represent outliers.

The alpha diversity analysis, i.e., species richness, revealed significant variability across the study zones (i.e., north, centre, and south). Species richness ranged from 4 to 56 species per site (Figure 4). One-way ANOVA revealed significant differences in species richness among the zones (F_2,23_ = 5.083, *p* = 0.015). Post hoc Tukey HSD comparisons indicated that species richness was significantly higher in the north zone (sites 1–8) compared to the centre zone (mean difference = 16.88, *p* = 0.024) and the south zone (mean difference = 15.25, *p* = 0.032), whereas there was no significant difference in richness between the south and centre zones (mean difference = 1.63, *p* = 0.955).

### 2.6. TWINSPAN

**Figure 5:**
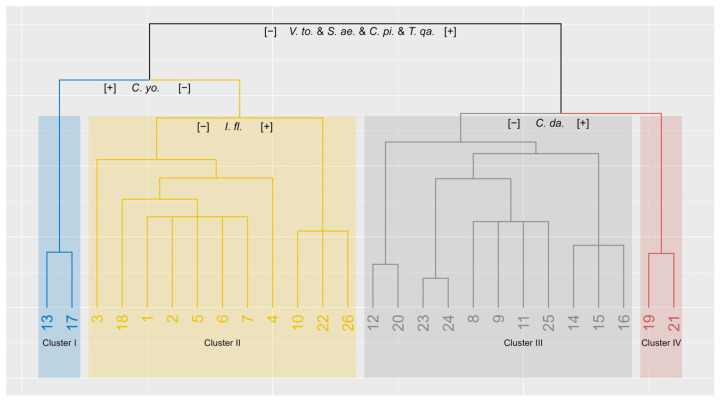
The TWINSPAN hierarchical clustering of the sampled depressions based on vegetation similarity.

TWINSPAN classified the depression sites into four distinct clusters, each defined by indicator species at successive splits in the classification hierarchy (Figure 5). The analysis used cut levels of 0, 2, 5, 10, and 20 to generate indicator pseudospecies based on their importance value index (IVI). The first major division (eigenvalue = 0.45) separated the sites based on the combined IVI of *Vachellia tortilis* (*V. to*), *Salvia aegyptiaca* L. (*S. ae*), *Convolvulus pilosellifolius* Desr. (*C. pi*), and *Tetraena qatarensis* (Hadidi) Beier & Thulin (*T. qa*). The first node was subsequently split into Clusters I and II using *Cyperus conglomeratus* (*C. yo*) as an indicator pseudospecies (eigenvalue = 0.566), while the second node was subsequently split into Clusters III and IV using *Cynodon dactylon* (L.) Pers. (*C. da*) as an indicator pseudospecies (eigenvalue = 0.402).

There was a clear difference in species composition and dominance among the four TWINSPAN-defined clusters (Table 2). Cluster I was characterised by the dominance of perennial grasses, particularly *Stipagrostis plumosa* Munro ex T.Anderson (IVI = 10.65) and the annual grass *Schismus arabicus* Nees (IVI = 9.66), indicating a grass-dominated community. Cluster II was dominated by a mix of woody perennials such as *Vachellia tortilis* (IVI = 8.42) and *Lycium shawii* Roem. & Schult. (IVI = 5.93), alongside the annual grass *Stipa capensis* (IVI = 6.46) and perennial herbs and grasses such as *Salvia aegyptiaca* and *Stipagrostis plumosa* (both IVI = 5.66), representing a shrubland habitat. Cluster III was dominated by woody perennials, most notably *Vachellia tortilis* (IVI = 31.07) and *Lycium shawii* (IVI = 11.65), with a minor presence of *Tetraena qatarensis* (IVI = 1.55). The herbaceous layer, comprising both annual herbs and grasses, was absent, indicating sparse overall vegetation cover. Cluster IV was characterised by the strong dominance of perennial grasses, particularly *Cynodon dactylon* (IVI = 38.35), accompanied by woody shrubs such as *Vachellia tortilis* (IVI = 12.72) and *Lycium shawii* (IVI = 5.72).

## 3. Discussion

### 3.1. Floristic Composition

The current study provides an update on the floristic diversity of the depression habitat in Qatar. The dominance of the Poaceae, Fabaceae, and Asteraceae families in Qatar’s depression habitat aligns with the findings from neighbouring arid regions [3,25,26,27]. For instance, in Saudi Arabia and Egypt, Poaceae, Fabaceae, and Asteraceae constitute the major components of the flora [25,26,27,28,29]. These families include species such as *Panicum turgidum*, *Vachellia tortilis*, and *Rhanterium epapposum* Oliv. which exhibit strong adaptations to drought stress [30]. Their resilience enables them to survive and thrive under the harsh environmental and physiological conditions characteristic of arid regions [30,31,32].

Our results showed that therophytes and chamaephytes are the most common floristic life forms in our study. This could be attributed to the irregular and highly variable nature of rainfall, which directly affects soil moisture, which in turn influences the vegetation patterns [2,33]. This pattern could also be attributed to the fact that grasses and herbaceous species typically produce a large number of small, persistent seeds that germinate rapidly following rainfall [18]. The dominance of therophytes and chamaephytes in Qatar’s depression reflects the region’s arid climate and limited rainfall [6,34,35]. Therophytes, which complete their life cycle within a single growing season, are well-adapted to environments with unpredictable water availability, allowing them to survive in Qatar’s arid conditions [2,6,25,33,34,35,36,37,38]. Chamaephytes, with their buds close to the soil surface, also adapt well to extreme conditions, such as high temperatures and wind exposure, which are common in Qatar’s landscape [24,25,39].

The variation in life form distribution may be influenced by local environmental factors, such as soil moisture and temperature gradients. For instance, the northern depressions have deeper soil layers compared to the southern and central depressions [2]. The higher therophytes in the northern region could be due to more pronounced seasonal variations in rains and specific silty soil that favour their growth [2]. Similarly, the distribution of chamaephytes across the northern and southern regions might reflect differences in microhabitats that support their survival [39,40]. Deep soil layers below 50 cm retain some moisture, sustaining deep-rooted perennials that survive prolonged droughts [2,6,41].

The current study recorded nine species of trees and wooden shrubs across Qatar’s depression habitat, including the introduced Vachellia nilotica and the invasive Prosopis juliflora. Earlier botanical work from Qatar reported a limited range of native tree phanerophytes, primarily comprising *Vachellia tortilis* (synonym: *Acacia tortilis*), *Vachellia flava* (Forssk.) Kyal. & Boatwr. (synonym: *Acacia ehrenbergiana*), *Lycium shawii*, *Ziziphus nummularia*, and *Prosopis cineraria* (L.) Druce [7]. Earlier, *Vachellia nilotica subsp. indica* (Benth.) Kyal. & Boatwr. (synonym: *Acacia nilotica subsp. indica*) and *Prosopis juliflora* (Sw.) DC. were documented as cultivated species commonly used in urban landscaping with limited distribution. However, in our study, we documented the presence of these two species within the natural habitats of the study area, which indicates the expansion in their distribution and highlights the growing impact of urbanisation on vegetation dynamics, particularly the distribution of invasive species into the northern depressions.

The location of Qatar within the extensive desert belt stretching from North Africa to Central Asia [2] explains the presence of the monoregional group with ca. 30 Saharo-Arabian species (Table 1). The Saharo-Arabian region is characterised by scarce vegetation that is shaped mainly by soil moisture [2,6,27]. The dominance of biregional chorotypes, particularly those involving the Saharo-Arabian region, reflects the influence of Qatar’s geographical position between several biogeographical zones, such as Irano-Turanian and Saharo-Arabian [2,6]. The prevalence of the Irano-Turanian/Saharo-Arabian and Mediterranean/Saharo-Arabian chorotypes highlights the region’s role as a transition zone between these major phytogeographical areas [27,42]. This transition facilitates the exchange of plant species, contributing to the diversity observed in Qatar’s flora.

The regional variations in chorotype distribution suggest that local environmental conditions and historical migration patterns have influenced the composition of plant communities across Qatar [7]. Species belonging to the Saharo-Arabian (SA) chorotype were more prevalent in the southern region of Qatar (20 species), followed by the northern depressions (15 species), and were least represented in the central depressions (8 species). This distribution suggests that southern Qatar offers more favourable ecological conditions for SA species due to its more arid conditions, whereas the northern and central regions receive relatively higher average annual rainfall [2,3]. Conversely, the species of biregional origin Saharo-Arabian/Irano-Turanian chorotypes showed a broader and more balanced distribution, with 21 species in the south, 20 in the north, and 14 in the central region. The relatively even distribution of these biregional species may reflect their ecological plasticity and ability to tolerate a wider range of edaphic and climatic conditions, which aligns with previous studies that have recognised the intersection of phytogeographical zones in Qatar [1,2,3].

### 3.2. Environmental Gradients and Vegetation Patterns

Latitude emerged as a significant spatial variable for vegetation composition based on the CCA, which reflects a north–south differentiation in environmental conditions across the study area, which, in turn, influences the distribution of species assemblages. This finding supports the previous works that showed a latitudinal variation in species composition in the vegetation pattern of Qatar [1,2,3]. The northern depressions are characterised by deep and fine soils resulting from the deposition of water-borne materials, whereas the southern depressions are mostly sandy with coarse substrates resulting from aeolian (wind-blown) deposition [2]. Further, the northern depressions are characterised by clay-rich soils that support nutrient-demanding woody species, which explains the dominance of species such as *Ziziphus nummularia* and *Vachellia tortilis* in our study [2]. The southern depressions, particularly sites 13 and 17, are characterised by sandy mounds that support distinct xerophytic plant communities [2,6] such as the *Panicum turgidum* plant community, which is typically found in the sandy depressions and plains across Qatar [2,4].

The CCA ordination showed a strong influence of edaphic factors, particularly nitrogen availability and clay content. Variations in soil nitrogen content can alter edaphic properties, thereby playing a crucial role in shaping plant community composition, distribution patterns, and vegetation productivity [43]. In arid environments, the interaction between soil pH and clay content plays a critical role in regulating nitrogen availability, thereby affecting the efficiency of plant nitrogen uptake, which is important to soil fertility [44].

Grazing is identified among the major factors shaping vegetation structure [45]. Our study confirms that grazing pressure has a broad impact on species distribution within the depression habitat (Figure 3). In heavily grazed areas, which are more concentrated at the central and southern depressions, browsing-tolerant shrubs such as *Vachellia tortilis* and *Lycium shawii* tend to dominate [2,46]. Overgrazing, particularly by camels, has resulted in the stunted growth of Vachellia trees, while individuals in undisturbed areas exhibit greater height and vitality [47,48]. Previous studies showed that grazing reduces plant diversity and alters community composition [49,50]. In our study, the central and southern sites showed a near-complete absence of annual ground cover, except in a few shallow depressions. The woody shrubs *Vachellia tortilis* and *Lycium shawii* were also heavily impacted, with no observed regeneration or new seedlings, indicating a disruption of their natural life cycle due to excessive grazing.

The depression habitat in Qatar plays a critical ecological role, supporting denser and more diverse vegetation compared to other desert habitats, such as the surrounding rocky ridges and plains [1,2]. Ephemeral species rapidly form a green cover following rainfall, especially in northern Qatar, where deeper soils and higher precipitation create favourable growth conditions [2,51]. Our study shows that the northern depressions, such as sites 2, 5, 6, and 7, exhibited higher species richness, probably due to greater soil depth and relatively higher rainfall [3]. In contrast, the southern and central depression sites, including sites 9, 11, 24, and 25, demonstrated lower species richness, largely attributed to overgrazing, which is a key factor contributing to the decline in species richness across several depression sites [47,50,52].

TWINSPAN grouped sites 13 and 17 within Cluster I, where *Vachellia tortilis* was almost absent. However, the presence of the psammophytic perennial sedge *Cyperus conglomeratus* in these sites reflects the sandy and saline edaphic conditions that characterise the southern depression habitat [4,53]. *Stipagrostis plumosa*, *Schismus arabicus*, and *Plantago boissieri* emerged as the most dominant species for this cluster (Table 2). The *S. plumosa* and *S. arabicus* grasses form localised patches in sandy shallow soils [54]. *Plantago boissieri* is an annual herb commonly associated with sandy plains and coastal lowland habitats [9,55]. Due to its high palatability, *Plantago boissieri* is subject to intense grazing pressure from both wild and domesticated herbivores, which has led to reduced cover in eastern Saudi Arabia and Qatar [55].

Cluster II, mostly representing the northern, southern, and central depressions, is dominated by *Vachellia tortilis*. In Qatar, this species is commonly found in depressions with deep, moist soils rich in organic carbon and available phosphorus [51]. This plant community exhibits a broad ecological range and can be subdivided into two main types [2]. The first type occurs in shallow depressions in the northern and central regions, often associated with *Lycium shawii* and *Ziziphus nummularia*. The second type is found in deeper, sandy depressions of southern Qatar, typically associated with *Tetraena qatarense* and a high cover of *Panicum turgidum*. The perennial herb Salvia aegyptiaca and the annual grass *Stipa capensis* are commonly distributed throughout the depressions, interspersed between the scattered woody shrubs, forming a characteristic plant community of these habitats. *Prosopis cineraria* and invasive *Prosopis juliflora* are among the documented species in this cluster. *P. cineraria* was first recorded in 2009 and is considered a rare species in Qatar [4]. Since then, *P. cineraria* has been widely used in vegetation restoration programmes across the country, driven by both governmental initiatives and individual efforts. *P. juliflora* was introduced for urban landscaping in the 1980s [2]. Since then, it has spread into depression habitats, resulting in a partial invasion in northern Qatar [56].

Cluster III encloses sites from the central and southern depressions and is characterised by the absence of annual herbs and other short-lived species, resulting in minimal ground-layer vegetation. The vegetation of this cluster is dominated by scattered woody trees and shrubs, primarily under stunted canopies of *Vachellia tortilis* and *Lycium shawii*. The near-total absence of annuals and short-lived perennials is likely a consequence of intensive overgrazing, which has led to the severe degradation of the herbaceous layer [46]. These communities are typical of shallow, clay-rich depressions with fine-textured sediments [3]. *Vachellia flava*, a characteristic tree species of this cluster, was recorded at sites 23 and 24. It typically occurs in slopes and wadi habitats that are characterised by sandy terrain and contain more clay [57]. In Qatar, *V. flava* is part of the tree layer in southern and central regions, where it prefers fine-textured soils [2]. The species often accumulates sandy mounds around its base, an adaptive feature that enhances its persistence in the depression habitat [2,7].

Cluster IV encloses sites 19 and 21, which are located in the central depressions. These sites are characterised by the presence of accumulated patches of *Cynodon dactylon* interspersed with scattered woody trees and shrubs such as *Vachellia tortilis* and *Lycium shawii*. *Cynodon dactylon* is the most dominant species in this cluster (Table 2). This grass forms localised patches in low-lying areas and along road edges where water tends to accumulate [58].

## 4. Materials and Methods

### 4.1. Study Area

**Figure 6:**
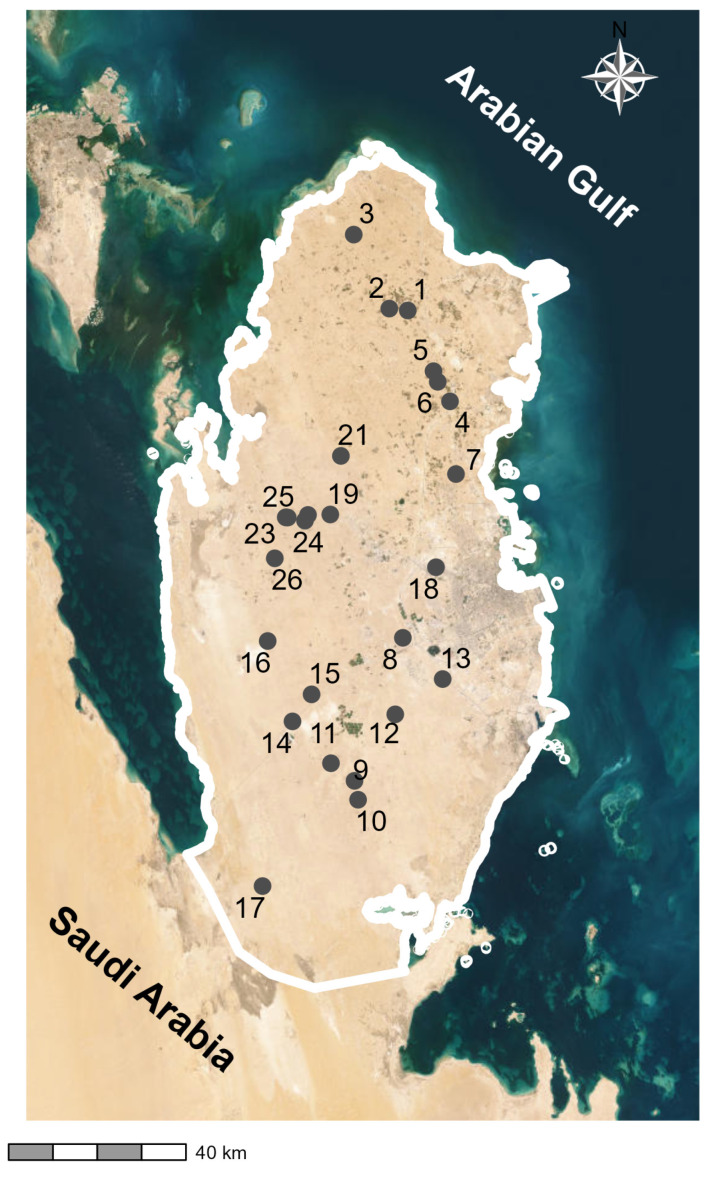
The distribution of the sampled depression sites. Where the numbers refer to the location of the depression sites.

Field surveys were conducted across the natural depression sites in Qatar between January 2021 and December 2022. The depression sites were selected to represent the latitudinal variability of the study area: north, south, and centre. Sites from 1 to 7 are the northern sites (Figure 6). The southern zone included sites 8 to 17, while the central zone encompassed sites 18 to 26 (Figure 6). As such, we represent the diversity of vegetation and environmental conditions within the country. At each site, sampling plots, each of 10 × 10 m, were determined according to the minimal area method to ensure adequate representation of the local vegetation [18]. The plots were established following the Relevé method [59] to ensure comprehensive coverage of different vegetation types, including trees, shrubs, herbs, and grasses. The plot location was selected to represent the variability within each depression, including both the central and peripheral zones. The number of plots per site ranged from four to ten depending on the size and heterogeneity of each site (Table S2). As such, we ensured that the sampling effort adequately reflected the extent and internal heterogeneity of each site and allowed for comparability among the depression sites.

Environmental parameters influencing vegetation patterns were recorded during field surveys, including main habitats and grazing pressure. Soil data, including organic carbon content (SOC), organic carbon stock (OCS), organic carbon density (OCD), pH, sand/silt/clay composition, bulk density, soil texture, and root depth, were retrieved from SoilGrids and Harmonized World Soil Database [60,61] at a fine spatial resolution of 250 m. Root depth is a categorical variable comprising four classes (1 to 4): very shallow (<10 cm), shallow (10–50 cm), moderate (50–100 cm), and deep (>100 cm) [60]. The shallow category (i.e., class 2) was not recorded in the study area, and therefore, the root depth was represented by three classes in our study. SOC refers to the concentration of carbon contained in organic matter within the soil and is a key indicator of soil fertility and microbial activity [61,62]. OCS represents the total amount of organic carbon stored in a vertical soil column to a defined depth (e.g., 0–30 cm). It integrates SOC concentration with bulk density and soil depth to quantify the total carbon stored per unit area [61], whereas OCD refers to the amount of organic carbon per unit volume of soil [61]. While conceptually similar to OCS, OCD does not account for depth and is instead a measure of the soil’s carbon storage capacity per unit volume [61].

It is evident that climatic factors influence the vegetation spatiotemporal pattern [63]. Therefore, climatic variables, specifically annual mean temperature and total annual precipitation since 2016, were extracted from the Chelsa dataset [64]. The environmental conditions (i.e., soil and climate) were summarised at the site level by calculating the median for the continuous variables and the mode for categorical variables across all the grid cells within a 1 km radius of each site centroid. As such, we ensured that the local variability is accounted for in the environmental data. The soil texture variable was mode-dominated, with 95% of the observations classified as loam. Therefore, it was excluded from the list of explanatory variables. Previous studies showed that the ecological patterns vary geographically across Qatar [2,3]. Therefore, we included two spatial variables, longitudinal and latitudinal, to represent the unmeasured environmental and anthropogenic gradients, such as oceanic influence in the north and east and land-use intensity in the south and west of the study area. These variables were the mean latitude and mean longitude of the depression sites. All the spatial data processing and analysis were performed using the “terra” R package [65].

### 4.2. Vegetation Sampling and Floristic Survey

For each plot, we recorded the number of individuals per species and the average foliage/canopy diameter (d) of individuals [66,67,68,69]. These metrics were then used to estimate the relative density, relative frequency, relative abundance, and relative cover for each species at the site level. Density, expressed as the number of individuals per square meter, was calculated by summing the total number of individuals of each species across all the plots within a given site and dividing this value by the total sampled area (i.e., the number of plots multiplied by the area of a single plot, 100 m^2^) [66,67,68,69,70]. Abundance was calculated by summing the total number of individuals observed for each species across all the plots and dividing this total by the number of plots in which the species was present [66,67,68,70]. Frequency was determined as the proportion of plots in which a given species occurred relative to the total number of plots surveyed at the site [66,68,70]. Plant cover was calculated using the average foliage diameter of a species using the following formula (1):

Plant cover = (3.14 × (d/2)^2^ × number of individuals of species)/Total area × 100 (1)

where “d” is the average diameter (cm) of individuals’ foliage.

To facilitate interpretation and standardise reporting, the vegetation parameters were then standardised by multiplying by 100, thereby expressing the vegetation parameters, such as species density, as the number of individuals per 100 m^2^ [66,68,70]. The relative vegetation parameters were calculated using standard formulas [66,67,68,70,71,72].

Relative Density: (Density of a species/Total density of all species) × 100(2)

Relative Frequency: (Frequency of a species/Total frequency of all species) ×100(3)

Relative Abundance: (Abundance of a species/Total abundance of all species) ×100(4)

Relative Cover: (Cover area of a species/Total cover area of all species) × 100(5)

These relative vegetation parameters were then used to compute the importance value index (IVI) for each species at each site [70,71] using the formula (6). The IVI provides an integrated measure of a species’ ecological importance within the plant community and is widely used to assess dominance and community structure [67,70,71,72,73].

IVI = (Relative Density + Relative Frequency + Relative Abundance + Relative Cover)/4(6)

### 4.3. Plant Life Form and Phytogeographical Analysis

Plant species were assigned to life form categories based on the modified Raunkiaer system [39,74], which categorises plants according to the position of their perennating buds. Additionally, the phytogeographical affinities for the recorded species were determined following [21,22] and other regional studies [31,37,39,57,75,76,77].

### 4.4. Multivariate Analyses

We applied advanced multivariate techniques to analyse the vegetation diversity and explore relationships between species composition and environmental variables. We formatted the field data into a site-by-species matrix to perform the multivariate analysis, using the IVI scores for a given species at a given site. The vegetation matrix (Table S3) was constructed at the site level, based on field data collected using multiple plots per site (4–10 quadrats of 100 m^2^ each). For each species within each site, we calculated the IVI by aggregating data across all the plots within that site and standardising them to a unit area of 100 m^2^ [66,68,70]. Further, we performed Canonical Correspondence Analysis (CCA) [78,79] to examine relationships between the vegetation patterns and environmental variables such as soil pH, sand content, organic carbon density, and grazing intensity [43,80]. We performed a permutation analysis (999 permutations) to evaluate whether the relationship between the species and environmental variables is statistically significant [81,82]. CCA and permutation analyses were performed using the “cca” and “anova.cca” functions implemented in the “vegan” R package [83].

Additionally, we performed a Two-Way Indicator Species Analysis (TWINSPAN) to classify the depression sites based on their similarity in species composition. We set the pseudospecies threshold at different IVI cutoff values of 0, 2, 5, 10, and 20 to create binary presence/absence variables for each species [81,82,84,85]. At each division step, diagnostic species were identified as those best differentiating the resulting clusters based on their consistent presence or absence above the pseudospecies thresholds [81,86]. TWINSPAN was performed using the “twinspan” function implemented in the “twinspan” R package [87].

Further, we calculated species alpha diversity, particularly species richness, for each site to identify the key sites with high floristic diversity [88]. We assessed the differences in species richness among the depression zones (north, south, and centre) by performing a one-way Analysis of Variance (ANOVA) [89] after verifying that the assumptions of normality and homogeneity of variance using the Shapiro–Wilk test (W = 0.95, *p* = 0.369) and Levene’s test (F = 2.75, *p* = 0.084), respectively, were met. Post hoc pairwise comparisons were performed using Tukey’s Honest Significant Difference (HSD) test with a 95% confidence level to identify specific differences between zone pairs.

## 5. Conclusions

This study provides a comprehensive assessment of the floristic diversity and environmental drivers shaping vegetation in Qatar’s depression habitat, which is a largely understudied habitat. A total of 139 plant species from 35 families were documented, including 81 perennials and 58 annuals. The dominance of therophytes and chamaephytes reflects the arid conditions of the region, while the presence of a limited number of phanerophytes, some of which are introduced or invasive, highlights the influence of anthropogenic factors on vegetation composition.

Chorological analysis identified three dominant chorotypes: monoregional Saharo-Arabian, Irano-Turanian/Saharo-Arabian, and Mediterranean/Saharo-Arabian, with the Saharo-Arabian element being the most prevalent across the study area. Multivariate analyses highlight the importance of soil properties and grazing pressure as the key factors in structuring plant communities. The northern depressions supported greater species richness and denser vegetation cover compared to the southern and central depressions, which were predominantly sandy and more heavily grazed and disturbed.

We showed that the depression habitat encompasses four distinct groups based on the similarity of species composition. Overall, this study fills the gap about the floristic diversity of depression habitat and demonstrates the combined impact of soil characteristics and grazing pressure on floristic diversity. Further work is required to assess the impact of global change, including climatic and land-use change, in order to establish a proactive conservation plan to ensure the sustainability of depression ecosystem services. We recommend establishing an effective monitoring programme to assess the distribution of invasive species and to inform the restoration plan.

## Figures and Tables

**Table 1:** Floristic chorotypes of plant species recorded in Qatar’s depression habitat illustrating their distribution across monoregional, biregional, and pluriregional phytogeographical groups.

Chorotype *	No. of Species per Category	Group	Presence of Floristic Category in North	Presence of Floristic Category in South	Presence of Floristic Category in Centre
SA + IR-TR	27	Biregional	20	21	14
SA	26	Monoregional	15	20	8
ME + SA	15	Biregional	11	6	5
SA + SU	9	Biregional	6	7	9
SU	6	Monoregional	4	2	5
PAN	5	Pluriregional	5	3	1
SA-SI + S-Z	4	Biregional	3	3	3
ME	4	Monoregional	4	1	2
ME + SA-SI	4	Biregional	3	2	1
COSM	4	Pluriregional	3	0	1
SA + IR-TR + SU	4	Pluriregional	4	2	2
SA-SI + IR-TR	3	Biregional	3	2	1
ME + SA + IR-TR	3	Pluriregional	2	3	1
ME + SA-SI + IR-TR	3	Pluriregional	2	1	0
SA-SI + IR-TR	3	Biregional	2	2	2
TR	3	Monoregional	2	0	2
ME + IR-TR	2	Biregional	2	0	0
S-Z + SA-SI	2	Biregional	2	1	1
Other minor chorotypes	1	Mixed	7	6	4

* floristic categories (the chorotypes) based on [21,22,23]: COSM = Cosmopolitan-; IR-TR = Irano-Turanian; ME = Mediterranean;; PAN = Pantropical; S-Z = Sudano-Zambezian; SA = Saharo-Arabian; SA-SI = Saharo-Sindian; SU = Sudanian; TR = Tropical; Other minor chorotypes that are represented by only a single species, include 13 floristic categories such as EU-SI = Euro-Siberian and PAL = Palaeotropical. A complete list of chorotypes is listed in Table S1.

**Table 2:** IVI values of dominant, co-dominant, and indicator species of the identified clusters.

Species	Life Form *	Cluster I	Cluster II	Cluster III	Cluster IV	All Sites
*Convolvulus pilosellifolius* Desr. (*C. pi*)	He	0	1.82	0.79	0	0.77
*Cynodon dactylon* (L.) Pers. (*C. da*)	Ge	0	2.4	0	38.35	4.08
*Cyperus conglomeratus* Rottb. (*C. yo*)	He	1.69	0	0	0	0.13
*Ifloga spicata* (Forssk.) Sch. Bip. (*I. fl*)	Th	0.35	0.68	0.25	0	0.42
*Lycium shawii* Roem. & Schult. (*L. sh*)	Ph	0	5.93	11.65	5.72	8
*Plantago boissieri* Hausskn. & Bornm. (*P. bo*)	Th	9.66	0	0	0	0.74
*Salvia aegyptiaca* L. (*S. ae*)	Ch	3.12	5.66	1.32	0	2.97
*Schismus arabicus* Nees (*S. ar*)	Th	9.67	0.28	0	0	0.86
*Stipa capensis* Thunb. (*S. ca*)	Th	2.08	6.46	0	0	2.89
*Stipagrostis plumosa* Munro ex T.Anderson (*S. pl*)	He	10.65	5.66	0	0	3.21
*Tetraena qatarensis* (Hadidi) Beier & Thulin (*T. qa*)	Ch	3.64	1.55	1.52	0	1.51
*Vachellia tortilis* (Forssk.) Galasso & Banfi (*V. to*)	Ph	0	8.42	31.07	12.72	18.18

* life forms following [24]—classification. Ch = chamaephytes; Ge = geophytes; He = hemicryptophytes; Ph = phanerophytes; Th = therophytes.

## Data Availability

The original contributions presented in this study are included in the Supplementary Materials.

## Supplementary Materials

The following are available online at https://www.mdpi.com/article/10.3390/plants14121807/s1, Table S1: List of the recorded species, taxonomic families, life forms, vegetation types, floristic categories, and importance value index (IVI). Table S2: Site-level data including the associated geographic coordinates, grazing, and soil variables. Table S3: Vegetation matrix (Site X Species) used to perform the multivariate analysis based on IVI values.
