# Supplementary material for: Vegetation Analysis and Environmental Relationships of Qatar’s Depression Habitat
Source: Plants (Basel). 2025 Jun 12;14(12):1807. doi: 10.3390/plants14121807 (PMC12197089; doi:10.3390/plants14121807)
Supplement: Supplementary file 1 [file plants-14-01807-s001.zip › Table S1.pdf]

Table S1: Species List with Ecological Attributes and Importance Value Index (IVI).

Species	Abbreviation	Family	Vegetation Type	Life Form	Floristic Categories (Chorotype)	IVI Zones			IVI Vegetation Clusters			
						North	South	Center	I	II	III	VI
Abutilon fruticosum **Guill. & Perr.**	*A. fr*	Malvaceae	Perennial	Ch	SU	0.16	0.00	0.33	0.00	0.00	0.00	1.03
Abutilon pannosum **(G.Forst.) Schltdl.**	*A. pa*	Malvaceae	Perennial	Ch	TR	0.00	0.00	0.15	0.00	0.14	0.09	0.00
Aeluropus lagopoides **(L.) Trin. ex Thwaites**	*A. la*	Poaceae	Perennial	He	SA-SI	0.00	0.58	0.00	1.06	0.00	0.00	0.00
Aerva javanica **(Burm.f.) Juss. ex Schult.**	*A. ja*	Amaranthaceae	Perennial	Ch	SA-SI+S-Z	0.78	0.10	0.08	0.00	0.04	0.00	0.00
Aizoon canariense **L.**	*A. ca*	Aizoaceae	Perennial	He	SA-SI+S-Z	0.56	0.17	1.27	0.00	1.31	1.30	0.00
Althaea ludwigii **L.**	*A. lu*	Malvaceae	Annual	Th	IR-TR +ME	0.00	0.00	0.00	0.00	0.35	0.00	0.00
Anabasis setifera **Moq.**	*A. se*	Amaranthaceae	Perennial	Ch	SA	0.05	0.11	0.00	0.24	0.00	0.00	0.00
Anagallis arvensis **caerulea Hartman**	*A. ar*	Primulaceae	Annual	Th	ME+IR-TR+EU-SI	0.51	0.00	0.00	0.00	0.03	0.00	0.00
Anastatica hierachuntica **Crantz**	*A. st*	Brassicaceae	Annual	Th	SA	0.06	0.21	3.98	0.00	3.15	0.49	0.00
Anchusa hispida **Forssk.**	*A. hp*	Boraginaceae	Annual	Th	SA-SI + IR-TR	0.20	0.00	0.10	0.00	0.04	0.06	0.00
Andrachne telephioides **L.**	*A. te*	Phyllanthaceae	Perennial	Ch	ME + SA-SI + IR-TR	0.32	0.00	0.00	0.00	0.08	0.00	0.00
Arnebia hispidissima **(Lehm.) A.DC.**	*A. rb*	Boraginaceae	Annual	Th	SA + SU	0.06	0.67	0.50	1.11	0.51	0.12	0.00
Asphodelus tenuifolius **Cav.**	*A. sp*	Asphodelaceae	Annual	Ge	SA+SU	0.00	0.00	0.36	0.00	0.24	0.00	0.00
Astragalus sieberi **DC.**	*A. si*	Fabaceae	Perennial	Ch	SA+ IR-TR	1.19	0.00	0.21	0.00	0.18	0.00	0.00
Astragalus tribuloides **Delile**	*A. tr*	Fabaceae	Annual	Th	SA-SI +IR-TR	0.30	0.00	0.14	0.00	1.10	0.00	0.00
Atractylis carduus **(Forssk.) C.Chr.**	*A. cu*	Asteraceae	Annual	Th	ME	0.15	0.00	0.19	0.00	0.29	0.00	0.00
Blepharis ciliaris **(L.) B.L.Burtt**	*B. ci*	Acanthaceae	Perennial	Ch	S-Z + SA-SI	0.00	0.37	3.20	0.00	1.89	1.09	0.00
Brachiaria eruciformis **(Sm.) Griseb.**	*B. ra*	Poaceae	Annual	Th	TR	0.00	0.00	0.29	0.00	0.00	0.00	0.93
Calendula arvensis **M.Bieb.**	*C. al*	Asteraceae	Annual	Th	ME + SA-SI + IR-TR	2.01	0.08	0.00	0.00	0.00	0.00	0.00

***Capparis spinosa* L.**	*C. sp*	Capparaceae	Perennial	Ch	ME + SA-SI + IR-TR	0.00	0.00	0.00	0.00	1.08	0.00	0.00
***Cenchrus ciliaris* L.**	*C. ci*	Poaceae	Perennial	He	SA+SU	0.00	0.00	0.14	0.00	0.00	0.00	1.01
***Centropodia forsskaolii* (Vahl) Cope**	*C. fo*	Poaceae	Perennial	Ge	SA + IR-TR	0.17	0.10	0.00	0.20	0.00	0.00	0.00
***Chenopodium murale* L.**	*C. mu*	Amaranthaceae	Annual	Th	COSM	0.03	0.00	0.00	0.00	0.10	0.00	0.00
***Chrozophora tinctoria* (L.) Raf.**	*C. ti*	Euphorbiaceae	Annual	Th	ME + SA	0.00	0.00	0.00	0.00	0.02	0.00	0.00
***Chrysopogon plumulosus* Hochst.**	*C. pl*	Poaceae	Perennial	Ch	SA + SU	0.00	1.44	1.03	4.40	0.42	0.48	0.00
***Cistanche tubulosa* (Schrenk) Wight**	*C. tu*	Orobanchaceae	Annual	Pr	ME+SA-SI+ IR-TR+S-Z	0.00	0.30	0.00	0.56	0.00	0.00	0.00
***Citrullus colocynthis* (L.) Schrad.**	*C. co*	Cucurbitaceae	Perennial	He	ME+ SA-SI+ IR-TR+ S-Z	0.61	0.77	0.52	0.00	1.43	0.95	0.00
***Cocculus pendulus* (J.R. & G. Forst.) Diels**	*C. pe*	Menispermaceae	Perennial	Ch	SU	0.00	0.00	3.55	0.00	1.41	1.10	3.06
***Convolvulus cephalopodus* Boiss.**	*C. ce*	Convolvulaceae	Perennial	Ch	SA	0.27	0.19	0.34	0.41	0.73	0.00	0.00
***Convolvulus fatmensis* Kunze**	*C. fa*	Convolvulaceae	Annual	Th	SA	0.07	0.00	0.00	0.00	0.12	0.00	0.00
***Convolvulus glomeratus* Choisy**	*C. gl*	Convolvulaceae	Perennial	Ch	SU	1.12	0.00	0.00	0.00	0.04	0.00	0.00
***Convolvulus pilosellifolius* Desr.**	*C. pi*	Convolvulaceae	Perennial	He	IR-TR + M	0.78	0.17	0.71	0.00	1.82	0.79	0.00
***Convolvulus prostratus* Forssk.**	*C. pr*	Convolvulaceae	Perennial	Ch	SA	0.66	1.96	2.55	1.12	0.94	4.93	1.87
***Corchorus depressus* (L.) Stocks**	*C. de*	Malvaceae	Perennial	Ch	ME+SA	0.16	3.60	2.17	0.00	1.92	5.98	0.00
***Corchorus trilocularis* L.**	*C. tr*	Malvaceae	Annual	Th	TR	0.18	0.00	0.00	0.00	0.04	0.00	0.00
***Cressa cretica* L.**	*C. cr*	Convolvulaceae	Perennial	He	ME+IR-TR	0.63	0.00	0.00	0.00	0.06	0.00	0.00
***Cullen plicatum* (Delile) C.H.Stirt.**	*C. pl*	Fabaceae	Perennial	Ch	SA + IR-TR	0.05	0.66	0.00	0.00	0.34	1.13	0.00
***Cuscuta chinensis* Lam. on Cullen**	*C. ch*	Convolvulaceae	Annual	Pr	SA-SI + S-Z	1.01	0.00	0.00	0.00	0.03	0.00	0.00
***Cymbopogon commutatus* (Steud.) Stapf**	*C. cy*	Poaceae	Perennial	Ch	SA	0.71	1.83	0.18	7.76	1.58	0.35	0.00
***Cynodon dactylon* (L.) Pers.**	*C. da*	Poaceae	Perennial	Ge	PAN	0.06	0.18	25.56	0.00	2.40	0.00	38.35
**Cynomorium coccineum L.**	*C. yn*	Cynomoriaceae	Perennial	Ge+Pr	ME + SA + IR-TR	0.00	0.20	0.00	0.41	0.11	0.00	0.00
***Cyperus conglomeratus* Rottb.**	*C. yo*	Cyperaceae	Perennial	He	SA	0.33	0.67	0.00	1.69	0.00	0.00	0.00
***Dichanthium annulatum* (Forssk.) Stapf**	*D. an*	Poaceae	Perennial	He	SA	0.90	0.00	0.00	0.00	0.60	0.00	0.00
***Dichanthium foveolatum* (Delile) Roberty**	*D. fo*	Poaceae	Perennial	He	TR+SA	0.09	0.00	0.00	0.00	2.78	0.00	0.00

***Dipterygium glaucum* Decne.**	*D. gl*	Capparaceae	Perennial	Ch	SA	0.32	0.09	0.00	0.18	0.19	0.00	0.00
***Emex spinosa* (L.) Campd.**	*E. sp*	Polygonaceae	Annual	Th	PAN	0.00	0.00	0.00	0.00	0.16	0.00	0.00
***Eragrostis barrelieri* Daveau**	*E. ba*	Poaceae	Annual	Th	ME + SA	0.05	0.12	2.18	0.00	0.00	0.33	2.70
***Eragrostis cilianensis* (All.) Janch.**	*E. ci*	Poaceae	Annual	Th	COSM	0.29	0.00	0.00	0.00	0.03	0.00	0.00
***Erodium laciniatum* (Cav.) Willd.**	*E. la*	Geraniaceae	Annual	Th	ME + SA	0.00	0.00	0.00	0.00	0.13	0.00	0.00
***Euphorbia chamaesyce* L.**	*E. ch*	Euphorbiaceae	Annual	Th	COSM	0.21	0.00	0.31	0.00	0.00	0.21	0.00
***Euphorbia granulata* Forssk.**	*E. gr*	Euphorbiaceae	Annual	Th	SU	0.54	0.00	0.40	0.00	0.26	0.00	3.20
***Fagonia indica* Burm.f.**	*F. in*	Zygophyllaceae	Perennial	Ch	SA +IR-TR	0.23	0.63	1.47	0.24	0.86	1.17	0.00
***Fagonia ovalifolia* Hadidi**	*F. ov*	Zygophyllaceae	Perennial	Ch	SA +IR-TR	0.06	0.15	0.29	0.00	0.40	0.19	0.00
***Fagonia bruguieri* DC.**	*F. br*	Zygophyllaceae	Perennial	Ch	SA +IR-TR	0.00	0.07	0.00	0.00	0.04	0.24	0.00
***Farsetia heliophila* Bunge ex Coss.**	*F. he*	Brassicaceae	Perennial	Ch	SA	0.24	0.18	0.00	0.61	0.00	0.00	0.00
***Filago desertorum* Pomel**	*F. de*	Asteraceae	Annual	Th	SA	0.20	0.00	0.00	0.00	0.11	0.00	0.00
***Glossonema varians* (Stocks) Benth. ex Hook.f.**	*G. va*	Apocynaceae	Perennial	Ch	IR-TR + SA	0.00	0.46	0.72	1.46	0.42	0.16	0.00
***Haloxylon persicum* Bunge**	*H. pe*	Amaranthaceae	Perennial	Ph	IR-TR + SA	0.17	0.19	0.00	0.58	0.00	0.00	0.00
***Haloxylon salicornicum* (Moq.) Bunge ex Boiss.**	*H. sa*	Amaranthaceae	Perennial	Ph	SA + IR-TR	0.03	1.22	0.22	3.51	0.05	0.40	0.00
***Haplophyllum tuberculatum* Juss.**	*H. tu*	Rutaceae	Perennial	Ch	SA + IR-TR	0.00	0.12	0.00	0.00	0.02	0.06	0.00
***Helianthemum kahiricum* Delile**	*H. ka*	Cistaceae	Perennial	Ch	SA	0.03	0.46	0.00	1.60	0.00	0.00	0.00
***Helianthemum ledifolium* (L.) Mill.**	*H. le*	Cistaceae	Annual	Th	ME + SA	0.50	0.00	0.00	0.00	0.02	0.00	0.00
***Helianthemum lippii* (L.) Dum.Cours.**	*H. li*	Cistaceae	Perennial	Ch	SA + IR-TR	0.15	0.89	0.08	2.44	0.65	0.00	0.00
***Heliotropium bacciferum* Forssk.**	*H. ba*	Boraginaceae	Perennial	Ch	SA-SI+S-Z	0.33	1.21	1.20	0.93	0.04	3.58	5.51
***Herniaria hirsuta* L.**	*H. hi*	Caryophyllaceae	Annual	Th	ME +SA-SI	0.18	0.00	0.00	0.00	0.18	0.00	0.00
***Herniaria hemistemon* J.Gay**	*H. he*	Caryophyllaceae	Annual	Th	ME +SA-SI	0.17	0.00	0.00	0.00	0.06	0.00	0.00
***Hippocrepis multisiliquosa* L.**	*H. mu*	Fabaceae	Annual	Th	IR-TR	0.00	0.00	0.00	0.00	0.06	0.00	0.00
***Ifloga spicata* (Forssk.) Sch.Bip.**	*I. fl*	Asteraceae	Annual	Th	ME + SA-SI	0.00	0.46	0.84	0.35	0.68	0.25	0.00
***Indigofera intricata* Boiss.**	*I. in*	Fabaceae	Perennial	Ch	SU	0.17	0.16	0.89	0.00	1.57	0.70	0.00
***Lasiurus scindicus* Henrard**	*L. sc*	Poaceae	Perennial	He	SA + SU	0.00	1.55	0.20	3.19	0.23	0.24	1.61
***Launaea angustifolia* (Desf.) Kuntze**	*L. an*	Asteraceae	Annual	Th	SA	0.20	0.19	0.00	0.36	0.00	0.00	0.00

**Launaea capitata (Spreng.) Dandy**	*L. ca*	Asteraceae	Perennial	Th	ME + SA-SI	0.00	0.19	0.00	0.00	0.13	0.00	0.00
***Launaea mucronata* (Forssk.) Muschl.**	*L. mu*	Asteraceae	Perennial	Th	SA-SI + IR-TR	0.20	0.40	0.00	0.98	0.00	0.00	0.00
***Launaea nudicaulis* (L.) Hook. f.**	*L. nu*	Asteraceae	Perennial	Th	SA-SI + IR-TR + S-Z	0.23	0.08	0.00	0.18	0.08	0.00	0.00
***Lepidium aucheri* Boiss.**	*L. au*	Brassicaceae	Annual	Th	IR-TR + SA	0.00	0.00	0.00	0.00	0.09	0.00	0.00
***Leptadenia pyrotechnica* (Forssk.) Decne.**	*L. py*	Apocynaceae	Perennial	Ph	SA + IR-TR	0.00	0.21	0.16	1.27	0.04	0.04	0.00
***Lotus garcinii* DC.**	*L. ga*	Fabaceae	Perennial	Ch	ME + SA	0.00	0.24	0.00	0.54	0.00	0.00	0.00
***Lotus halophilus* Boiss. & Spruner**	*L. ha*	Fabaceae	Annual	Th	ME	5.55	0.49	0.00	0.89	0.00	0.00	0.00
***Lycium shawii* Roem. & Schult.**	*L. sh*	Solanaceae	Perennial	Ph	SA+SU + IR-TR	0.22	4.66	7.18	0.00	5.93	11.65	5.72
***Malva parviflora* L.**	*M. pa*	Malvaceae	Annual	Th	PAN	0.27	0.13	0.00	0.00	0.24	0.00	0.00
***Medicago laciniata* (L.) Mill.**	*M. la*	Fabaceae	Annual	Th	ME + SA	0.00	0.13	0.26	0.38	0.35	0.00	0.00
***Moltkiopsis ciliata* (Forssk.) I.M. Johnst.**	*M. ci*	Boraginaceae	Perennial	Ch	SA	0.00	0.29	0.00	0.57	0.00	0.00	0.00
***Monsonia nivea* (Decne.) Webb**	*M. ni*	Geraniaceae	Perennial	Ch	SA	0.00	1.08	0.00	2.03	0.00	0.00	0.00
***Neurada procumbens* L.**	*N. pr*	Neuradaceae	Annual	Th	SA	0.20	3.71	0.46	7.33	0.00	1.15	0.00
***Ochradenus baccatus* Delile**	*O. bc*	Resedaceae	Perennial	Ph	SA + IR-TR	0.00	0.21	1.54	0.00	0.98	1.03	0.00
***Ochthochloa compressa* (Forssk.) Hilu**	*O. co*	Poaceae	Perennial	He	SA	0.00	3.53	0.00	0.00	0.00	3.09	0.00
***Ogastemma pusillum* (Coss. & Durand ex Bonnet & Baratte) Brummitt**	*O. pu*	Boraginaceae	Annual	Th	SA	0.14	0.56	0.00	0.00	0.00	0.22	0.00
***Pallenis hierochuntica* (Michon) Greuter**	*P. hi*	Asteraceae	Annual	Th	SA	0.00	0.00	0.00	0.00	0.04	0.00	0.00
***Panicum turgidum* Forssk.**	*P. tu*	Poaceae	Perennial	He	SA + SU	0.00	0.67	0.28	1.71	0.32	0.00	0.00
***Panicum coloratum* L.**	*P. cl*	Poaceae	Perennial	He	PAL	0.00	0.26	0.21	0.00	0.24	0.33	0.00
***Pennisetum divisum* (J.F. Gmel.) Henrard**	*P. di*	Poaceae	Perennial	He	SA + IR-TR	0.46	0.10	0.00	0.00	0.00	0.11	0.00
***Pentanema divaricatum* Cass.**	*P. dv*	Asteraceae	Annual	Th	ME + SA	0.30	0.00	0.00	0.00	0.26	0.00	0.00
***Phalaris minor* Retz.**	*P. mi*	Poaceae	Annual	Th	ME+IR-TR	0.18	0.00	0.00	0.00	0.20	0.00	0.00
***Plantago amplexicaulis* Cav.**	*P. la*	Plantaginaceae	Annual	Th	SA-SI +IR-TR	0.00	0.00	0.00	0.00	0.07	0.00	0.00

***Plantago boissieri* Hausskn. & Bornm.**	*P. bo*	Plantaginaceae	Annual	Th	SA	0.41	6.00	0.00	9.66	0.00	0.00	0.00
***Plantago ciliata* Desf.**	*P. ci*	Plantaginaceae	Annual	Th	ME+SA-SI+ IR-TR	0.16	0.21	0.00	0.58	1.59	0.00	0.00
***Plantago coronopus* L.**	*P. co*	Plantaginaceae	Annual	Th	ME + SA + EU-SI	0.03	0.00	0.00	0.00	0.06	0.00	0.00
***Plantago lanceolata* L.**	*P. nl*	Plantaginaceae	Annual	Th	ES + ME+ IR-TR	8.51	0.00	0.00	0.00	0.02	0.00	0.00
***Plantago ovata* Forssk.**	*P. ov*	Plantaginaceae	Annual	Th	SA+IR-TR	0.00	0.00	0.39	0.00	3.50	0.42	0.00
***Polycarpaea repens* (Forssk.) Asch. & Schweinf.**	*P. re*	Caryophyllaceae	Perennial	He	SA	0.11	0.44	0.25	0.85	0.19	0.00	0.00
***Polycarpaea robbairea* (Kuntze) Greuter & Burdet**	*P. rr*	Caryophyllaceae	Perennial	Ch	SA	0.67	0.00	0.00	0.00	0.06	0.00	0.00
***Prosopis cineraria* (L.)Druce**	*P. ci*	Fabaceae	Perennial	Ph	SA + IR-TR + SU	1.88	0.00	0.00	0.00	0.86	0.00	0.00
***Prosopis juliflora* (Sw.)DC.**	*P. ju*	Fabaceae	Perennial	Ph	PAN	0.27	0.05	0.00	0.00	1.32	0.11	0.00
***Pulicaria gnaphalodes* (Vent.) Boiss.**	*P. gn*	Asteraceae	Perennial	Ch	SA + IR-TR	0.00	0.54	0.21	1.75	0.33	0.00	0.53
***Pulicaria sicula* (L.) Moris**	*P. sc*	Asteraceae	Perennial	Ch	ME	1.68	0.00	0.14	0.00	0.08	0.00	0.00
***Pulicaria undulata* (L.) C.A.Mey.**	*P. ud*	Asteraceae	Perennial	Ch	SA + SU	0.15	1.82	2.67	0.55	2.95	3.87	2.33
***Reichardia tingitana* (L.) Roth**	*R. ti*	Asteraceae	Annual	Th	ME + SA	0.00	0.00	0.10	0.00	0.05	0.06	0.00
***Reseda muricata* C.Presl**	*R. mu*	Resedaceae	Annual	Th	ME + SA	0.00	0.22	0.00	0.62	0.00	0.00	0.00
***Rhanterium epapposum* Oliv.**	*R. ep*	Asteraceae	Perennial	Ch	SA	0.39	0.29	0.00	1.35	0.00	0.00	0.00
***Rhynchosia minima* (L.) DC.**	*R. mi*	Fabaceae	Perennial	Ch	PAN	0.19	0.00	0.00	0.00	0.14	0.00	0.00
***Rostraria pumila* (Desf.) Tzvelev**	*R. pu*	Poaceae	Annual	Th	SA+IR-TR	0.19	0.00	0.00	0.00	0.12	0.00	0.00
***Rumex vesicarius* L.**	*R. ve*	Polygonaceae	Annual	Th	ME	0.06	0.00	0.00	0.00	0.07	0.00	0.00
***Salsola imbricata* Forssk.**	*S. im*	Amaranthaceae	Perennial	Ch	SA+IR-TR	0.00	0.00	0.24	0.00	0.08	0.37	0.00
***Salsola vermiculata* L.**	*S. ve*	Amaranthaceae	Perennial	Ch	ME + SA+ IR-TR	2.86	0.09	0.00	0.19	0.00	0.00	0.00
***Salvia aegyptiaca* L.**	*S. ae*	Lamiaceae	Perennial	Ch	SA + ME	0.07	3.68	1.29	3.12	5.66	1.32	0.00
***Savignya parviflora* (Delile) Webb**	*S. pa*	Brassicaceae	Annual	Th	SA-SI+IR-TR	0.21	0.38	0.00	1.25	0.13	0.00	0.00
***Schismus arabicus* Nees**	*S. ar*	Poaceae	Annual	Th	SA+ IR-TR	1.71	3.88	0.00	9.67	0.28	0.00	0.00
***Sclerocephalus arabicus* Boiss.**	*S. cl*	Brassicaceae	Annual	Th	SA	0.35	1.98	2.58	0.80	2.34	6.19	0.00
***Scrophularia deserti* Delile**	*S. de*	Scrophulariaceae	Perennial	Ch	SA+IR-TR	0.00	0.00	0.23	0.00	0.26	0.15	0.00

***Seetzenia lanata* (Willd.) Bullock**	*S. la*	Zygophyllaceae	Perennial	Ch	SA+IR-TR	0.08	0.09	0.00	0.00	0.00	0.00	0.00
***Senna italica* Mill.**	*S. it*	Fabaceae	Perennial	Ch	SA + SU	0.03	0.47	0.76	0.00	0.14	0.75	5.13
***Sonchus oleraceus* (L.) L.**	*S. ol*	Asteraceae	Annual	Th	COSM	6.19	0.00	0.00	0.00	0.02	0.00	0.00
***Spergula fallax* (Lowe) E.H.L.Krause**	*S. fa*	Caryophyllaceae	Annual	Th	ME + SA	11.72	0.00	0.00	0.00	2.64	0.00	0.00
***Spergularia diandra* (Guss.) Boiss.**	*S. di*	Caryophyllaceae	Annual	Th	ME + SA	14.76	0.00	0.00	0.00	5.05	0.00	0.00
***Stipa capensis* Thunb.**	*S. ca*	Poaceae	Annual	Th	SA + IR-TR	5.53	0.82	0.00	2.08	6.46	0.00	0.00
***Stipagrostis plumosa* Munro ex T.Anderson**	*S. pl*	Poaceae	Perennial	He	SA + IR-TR	0.60	14.25	0.00	10.65	5.66	0.00	0.00
***Tamarix aphylla* (L.) Karst.**	*T. ap*	Tamaricaceae	Perennial	Ph	SA + IR-TR + SU	1.20	0.10	0.00	0.26	0.32	0.00	0.00
***Tetraena qatarensis* (Hadidi) Beier & Thulin**	*T. qa*	Zygophyllaceae	Perennial	Ch	SA	0.84	1.90	0.58	3.64	1.55	1.52	0.00
***Tetraena simplex* (L.) Beier & Thulin**	*T. si*	Zygophyllaceae	Annual	Th	SA + IR-TR	0.00	0.27	0.74	0.56	0.78	0.00	1.25
***Tribulus macropterus* Boiss.**	*T. ma*	Zygophyllaceae	Perennial	Ch	SA + IR-TR	0.00	0.27	0.00	0.53	0.00	0.00	0.00
***Tribulus terrestris* L.**	*T. te*	Zygophyllaceae	Perennial	Ch	ME + SA + IR-TR	4.20	0.13	0.16	0.00	0.00	0.14	1.30
***Trigonella anguina* Delile**	*T. ng*	Fabaceae	Annual	Th	ME + SA	0.80	0.00	0.00	0.00	2.04	0.00	0.00
***Trigonella stellata* Forssk.**	*T. st*	Fabaceae	Annual	Th	SA	0.00	0.00	0.00	0.00	0.91	0.00	0.00
***Vachellia flava* (Forssk.) Kyal. & Boatwr.**	*V. eh*	Fabaceae	Perennial	Ph	SA+SU	0.12	2.77	3.26	1.59	0.18	4.65	3.64
***Vachellia nilotica* (L.) P.J.H.Hurter & Mabb.**	*V. ni*	Fabaceae	Perennial	Ph	S-Z + SA-SI	4.59	0.00	0.00	0.00	0.06	0.00	0.00
***Vachellia tortilis* (Forssk.) Galasso & Banfi**	*V. to*	Fabaceae	Perennial	Ph	SU	0.00	18.54	12.55	0.00	8.42	31.07	12.72
***Zilla spinosa* (L.) Prantl**	*Z. sp*	Brassicaceae	Perennial	Ch	SA + IR-TR	3.44	0.06	0.16	0.00	0.04	0.20	0.00
***Ziziphus nummularia* (Burm.f.) Wight & Arn.**	*Z. nu*	Rhamnaceae	Perennial	Ph	SA + IR-TR + SU	0.00	1.30	7.11	0.00	3.19	4.58	8.12
***Ziziphus spina-christi* (L.) Desf.**	*Z. sc*	Rhamnaceae	Perennial	Ph	SA + SU + IR-TR	0.00	0.00	0.13	0.00	0.00	0.57	0.00

Vegetation type: Annual / Perennial. Life form using Raunkiaer's (1934) classification. Ch = chamaephytes; Cr = cryptophytes; Ge = geophytes; He = hemicryptophytes; Pr = parasites; Ph = phanerophytes; Th = therophytes. Floristic categories (the Chorotypes) based on Zohary (1973) and Eig (1931): COSM = Cosmopolitan; EU-SI = Euro-Siberian; IR-TR = Irano-Turanian; ME = Mediterranean; PAL = Palaeotropical; PAN = Pantropical; S-Z = Sudano-Zambezian; SA = SaharoArabian; SA-SI = Saharo-Sindian; SU = Sudanian; TR = Tropical.

Raunkiaer, C. The Life Forms of Plants and Statistical Plant Geography; Being the Collected Papers of C. Raunkiær. 1934. Available online: https://archive.org/details/in.ernet.dli.2015.271790 (accessed on 24 September 2024).
